# The potential of genome-oriented blood culture surveillance of vancomycin-resistant enterococcus faecium (VRE) to mirror local VRE epidemiology: a retrospective analysis and systematic comparison of VRE blood culture and VRE first patient isolates

**DOI:** 10.1186/s12879-025-12183-9

**Published:** 2025-11-28

**Authors:** Aila Caplunik-Pratsch, Bärbel Kieninger, Stefan Hansch, Anca Rath, Jürgen Fritsch, Anja Eichner, Frank Hanses, Wulf Schneider-Brachert, Florian Hitzenbichler

**Affiliations:** Department of Infection Prevention and Infectious Diseases, University Hospital Regensburg, Franz-Josef-Strauss-Allee 11, 93053 Regensburg, Germany

**Keywords:** Genomic epidemiology, Hospital transmission, Genome-oriented surveillance, Bloodstream infections, Core genome multilocus sequence typing (cgMLST), VRE

## Abstract

**Objectives:**

To evaluate whether vancomycin-resistant *Enterococcus faecium* (VRE) blood culture isolates reflect the broader hospital VRE epidemiology and to investigate the population structure of VRE in the context of rising bloodstream infection (BSI) rates at our institution.

**Methods:**

Whole genome sequencing (WGS) of VRE BSI isolates and all VRE first annual patient isolates (screening and clinical specimens) at a university tertiary care hospital from 2018 to 2021 was performed. Isolates were analysed using multi-locus sequence typing (MLST), core-genome (cg) MLST, and cluster analysis based on pairwise allelic differences.

**Results:**

From 2018 to 2021, 128 patients had VRE BSI and 218 had VSE (vancomycin-susceptible *Enterococcus faecium*) BSI, however, VRE became dominant in 2021 (2018: 53 vs. 21; 2021: 42 vs. 48). Concurrently, VRE incidence at our institution rose from 2.7 to 4.3 per 1000 patient days. WGS was performed for 125/128 VRE BSI isolates (97.7%) and 1175/1534 first annual isolates (76.6%). Distribution of complex types (CTs) within VRE blood culture isolates and first annual isolates was generally similar: ST80/CT1065, ST117/CT5130, ST1299/CT1903 and ST117/CT71 were detected most often both in BSI and among first annual isolates (26.5% vs. 26.6%; 14.4% vs. 10.8%; 12.0% vs. 13.5%; 10.4% vs. 13.3%, respectively). Eleven large clusters comprising 10 or more isolates were identified within the representative isolate cohort (a curated dataset including all sequenced blood culture isolates and non-redundant first annual patient isolates), consistent with clonal expansion of successful lineages, likely driven at least in part by in-hospital transmission events, but also possibly by repeated introductions of circulating clones, contributing to the rise in VRE incidence.

**Conclusions:**

Performing WGS on both VRE blood culture and first annual isolates points to several outbreaks as the reason behind the increase in VRE BSI rate. Our results demonstrate that sequencing VRE blood culture isolates alone roughly reflects the distribution of all VRE CTs, making it a pragmatic and resource-efficient approach for obtaining valuable information about the epidemiology of VRE within a hospital.

**Clinical trial:**

Not applicable.

**Supplementary information:**

The online version contains supplementary material available at 10.1186/s12879-025-12183-9.

## Introduction

Infections with Vancomycin-resistant *Enterococcus faecium* (VRE), particularly bloodstream infections (BSIs), pose a significant challenge in hospital settings, contributing to substantial morbidity and mortality [1, 2]. In Germany, data from the Antibiotic Resistance Surveillance Program (ARS) of the Robert Koch Institute for the period from 2017 to 2021 show an increase in the absolute number of VRE BSIs, peaking in 2021. However, the rate of VRE compared to vancomycin-susceptible *Enterococcus faecium* (VSE) was highest in 2019 at 25% and has decreased to 20% since then [3]. In contrast to the mixed national trends, our university tertiary care hospital experienced a marked increase in VRE BSIs between 2018 and 2021, with VRE BSIs surpassing VSE BSIs in 2021.

We considered several possible contributors to this local rise, including nosocomial transmission events, clonal expansion of endemic strains, or repeated independent introductions of high-risk VRE clones. While specific outbreaks were known to have occurred during the study period, detailed spatiotemporal analysis was beyond the scope of this work.

Against this background, we pursued two key aims: (1) to evaluate whether sequencing of VRE blood culture isolates alone can adequately reflect the broader VRE epidemiology within the hospital (a “pars pro toto” approach), and

(2) to investigate the population structure and temporal clustering of VRE isolates in the context of rising BSI numbers at our institution.

To address these aims, we performed whole genome sequencing (WGS) on all available VRE blood culture isolates and first annual VRE patient isolates (including both screening and clinical specimens) collected from 2018 to 2021.

To the best of our knowledge, this is the first study to systematically compare data from VRE blood culture isolates with first annual VRE patient isolates, including a significant percentage of screening isolates. This approach allows us not only to analyze the specific situation at our hospital but also to explore whether blood culture isolates alone adequately represent hospital-wide VRE epidemiology. Blood culture isolates reflect clinically significant infections, are routinely collected with high diagnostic accuracy, and are often prioritized in molecular surveillance programs. Assessing their representativeness therefore offers valuable insight into the practical utility of performing WGS on VRE blood culture isolates only.

This question is particularly relevant in the context of recent technological advances in genomic surveillance, which carry enormous potential to improve infection control in healthcare systems [4]. However, due to financial constraints, even in high-income countries, this technology is implemented in only a minority of hospitals. Evaluating whether sequencing a targeted subset of clinical samples can effectively capture epidemiological trends is thus of high practical importance.

## Material and methods

The ethics committee of the University of Regensburg (approval number 22–3027-104; date: 2/8/2022) approved the study protocol.

### Patient identification

Only isolates identified as belonging to the species *Enterococcus faecium (E.faecium)* were included in this study; therefore, the term “VRE” refers specifically to vancomycin-resistant *E. faecium* throughout the manuscript. All patients with a positive VRE blood culture from 2018 to 2021 were retrieved retrospectively from our hospital database. The first patient VRE blood culture isolate was included in the study.

First annual patient isolates: The first VRE isolate for each calendar year per patient at our institution was identified in the laboratory database. This included both **screening isolates** (obtained from rectal swabs) and **clinical isolates**, which were defined as all isolates derived from diagnostic specimens submitted in the context of clinical symptoms (e.g., wound swabs, urine, respiratory samples, or blood cultures). If a blood culture was the first isolate of the year, it was included here. If the first isolate was unavailable, we included the next available isolate of the same year. To account for possible strain changes over time within the same patient [5], we included the first VRE isolate of each study year per patient, not only the first-ever isolate. VRE screening at our hospital was performed as part of routine infection control measures. Contact patients of identified VRE carriers were consistently screened. In addition, targeted routine screening protocols were in place in certain high-risk departments and wards, such as for all patients undergoing hematopoietic stem-cell transplantation and in selected intensive care units (ICUs). During periods of increased VRE incidence or suspected outbreaks, intensified screening strategies were implemented in affected wards based on recommendations from the hospital’s infection control unit. The extent and frequency of screening varied over time and between departments, reflecting the reactive and pragmatic nature of infection control efforts rather than a standardized research protocol. All screenings were performed using rectal swabs. It is to mention that the WGS data of the first patient isolates of the year 2020 have already been published [6].

### Laboratory procedures

Screening specimens (rectal swabs) were collected using standard transport swabs and inoculated into BD Enterococcosel Broth (Becton Dickinson) supplemented with 6 µg/mL vancomycin and incubated for 24 hours at 36 ± 1 °C. Broth cultures were examined for esculin hydrolysis, and if positive (black discoloration), DNA was extracted and subjected to PCR for species identification and to detect the presence of *vanA* and *vanB* genes.

Clinical isolates were obtained from routine diagnostic specimens (e.g., blood cultures, urine, wound swabs) submitted by the treating clinical departments, using standard collection and transport methods. Specimens were plated on Columbia agar with 5% sheep blood (Oxoid), incubated under aerobic conditions, and colonies were identified using matrix-assisted laser desorption ionization time-of-flight mass spectrometry (MALDI-TOF MS; Bruker microflex, Mannheim, Germany). Antimicrobial susceptibility testing was performed using the BD Phoenix™ system (Becton Dickinson), and results were interpreted using EUCAST clinical breakpoints. Vancomycin resistance was confirmed by PCR detection of *vanA* or *vanB* genes.

All VRE isolates included in the study were stored at −80 °C in cryotubes prior to DNA extraction.

The available VRE samples for this study were thawed and grown on blood agar plates (Oxoid/Thermo Fisher Diagnostics GmbH, Wesel, Germany). Species identification was conducted using MALDI-TOF MS. Thereafter, specific polymerase chain reaction (PCR) for *vanA* and *vanB* was performed [7].

WGS was performed by extraction of DNA from pure 24-hours-old cultures with a QIAmp DNA Mini Kit (Qiagen Diagnostic GmbH, Germany), DNA concentration and quality was measured with Qubit (dsDNA HS array kit, Thermo Fisher Scientific, Germany). Sequencing libraries were created using the Nextera XT library Prep Kit (Illumina, USA). Sequencing was done on either a MiniSeq or Next-Seq D×550 (Illumina, USA), acquiring 2x150bp reads using either a high output (MiniSeq) or mid-output cassette (NextSeq DX50).

### Analysis of WGS data

De novo genome assembly of Illumina paired-end reads was performed using SKESA, version 2.4.0, integrated in SeqSphere+ version 9.0.1 (Ridom GmbH, Münster, Germany). Sequencing reads with a mean assembled coverage depth of 118,5x (range 28–192) and a mean percentage of good targets of 98.92 (range 90.1–99.6) were further analysed by multi-locus sequence typing (MLST) [8] and core-genome (cg) MLST [9] using SeqSphere+, which is based on 1,423 core genome targets. Allele calling was performed using SeqSphere+ default thresholds, requiring ≥90% nucleotide identity and ≥99% alignment coverage; targets not meeting these criteria were excluded from the cgMLST profile [10].

During minimum spanning tree construction and allelic distance calculation, missing loci were handled by pairwise ignoring missing values; that is, distances were calculated based only on loci present in both isolates.

The NCBI AMRFinderPlus software (version 3.2.3; database version 2019–10-30.1), as implemented in SeqSphere+, was used to identify *van* resistance genes in silico. Based on a distance matrix or respectively on neighbor joining trees created in SeqSphere+, we visualized genetic relationships between bacterial isolates. Annotations of the figures were done with itol (interactive tree of life) version 6.9.1 [11].

Affiliation to a cluster was defined as genotypes with a maximum difference of three alleles in pairwise cgMLST comparison, in accordance with thresholds previously used to identify likely recent transmission events in hospital settings [6, 12]. Clusters were defined using a single-linkage approach, whereby each isolate in a cluster was connected to at least one other isolate in the same cluster by a maximum of three allelic differences. This approach does not require that all pairwise distances within a cluster fall below the threshold.

### Representative isolate cohort

To create a representative isolate cohort of the hospital-wide VRE epidemiology we included all sequenced VRE blood culture and first patient isolates into one analysis; several isolates of one patients could be included if they were genetically distant from each other: If blood culture and annual first patient isolates were from the same complex type (CT), only the blood culture isolate was included. If no blood culture isolate was available, the earliest annual first patient isolate was used. More than one first annual isolate per patient could be in included in case of differing CTs. After cluster analysis, we ensured that clusters containing isolates from more than one CT did not contain more than one isolate from the same patient. The aim was to ensure that, in the rare cases where multiple isolates from the same patient with differing CTs but minimal allelic differences were included, it would not lead to the creation of artificial clusters. Only one cluster (ST80/CT1065 and CT3243) required exclusion of an additional isolate to maintain representativeness. Illustration of the inclusion/exclusion criteria of isolates for the creation of the representative isolate cohort in suppl Table 1.

### Pairwise distance analysis

Pairwise cgMLST allelic distances were calculated for all isolates of the representative isolate cohort. Distances were visualized as histograms to assess the distribution across the entire dataset (suppl Fig. 2), within the four largest clusters (suppl Fig. 3), and stratified by sequence type and cluster membership (suppl Fig. 4 and 5).

### Statistical methods

**Fig. 1:**
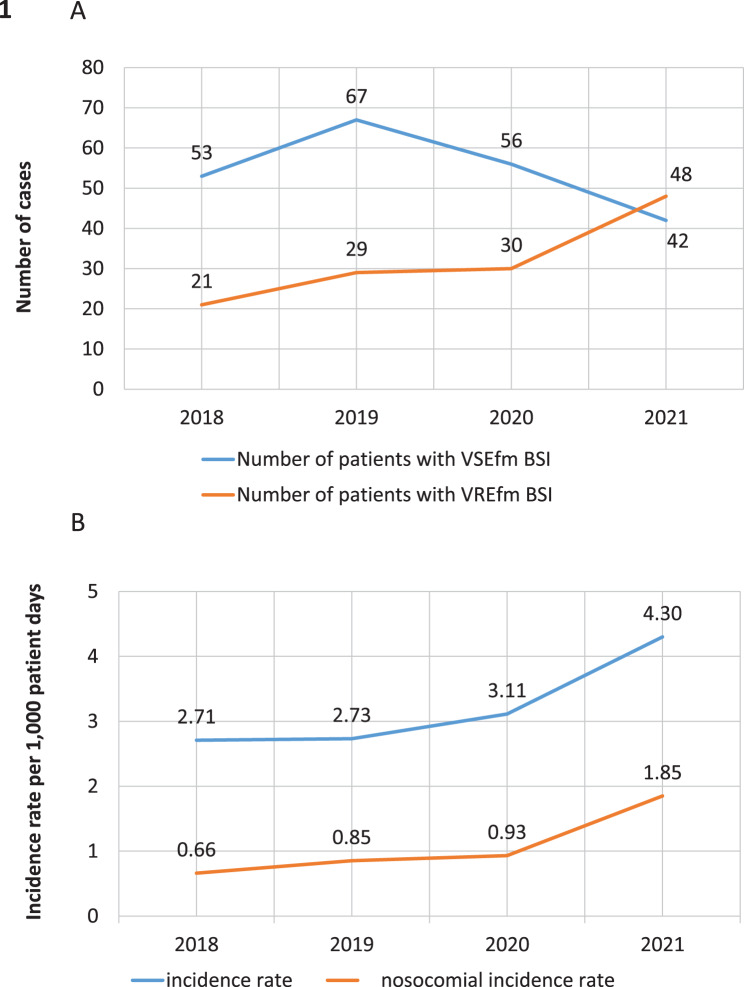
VRE epidemiological data from the university hospital regensburg from 2018 to 2021 **A**. VRE (red line) and VSE (blue line) bacteremia cases during the study period. **B**. VRE and nosocomial VRE incidence rate per 1,000 patient days. There was a steady increase in VRE BSI rates during the study period which is mirrored in the VRE incidence rate

The VRE incidence rates presented in Fig. 1b were calculated by the hospital’s infection control team as part of routine infection surveillance. Rates were expressed per 1,000 patient days and defined as follows: Overall VRE incidence rate = (Number of VRE-positive hospital admissions per year ÷ Total patient days per year) × 1,000

Nosocomial VRE incidence rate = (Number of hospital admissions with VRE detected ≥3 days after admission per year ÷ Total patient days per year) × 1,000

Each VRE-positive hospital admission was counted as one case. To avoid multiple counting of closely spaced readmissions, a 90-day interval rule was applied: repeated admissions of the same patient were only counted as new cases if more than 90 days had passed since the last VRE detection. Both colonization and infection were included.

Means and standard deviations were calculated for all ratio variables, while absolute frequencies and percentages were calculated for all nominal variables.

To ascertain whether there are scientific indications of differences in the distribution of the most common CTs and *van* resistance genes between VRE first patient isolates and VRE blood culture isolates, Fisher’s exact test was conducted. As a preliminary investigation into potential scientific causes with a limited number of cases, the significance level α was set at 0.05, without correction for multiple testing. The statistical analyses were conducted using the R statistical computing software.

## Results

### Epidemiological data at our institution

In 2018, the VSE/VRE ratio in BSIs was greater than 2 (53 vs. 21). During the following years, VRE BSIs gradually increased until, in 2021, VRE became more frequent (42 vs. 48, Fig. 1a). Simultaneously, the VRE incidence rate at our institution grew constantly over the study period and peaked in 2021 at 4.3 per 1,000 patient days (Fig. 1b).

### VRE first patient isolates

Within the study period, 1,534 first annual VRE patient isolates were identified, of these 1,175 were available for WGS (76.6%; rate ranging from 51% to 91% across the years, suppl Table 2). Among the 1,175 first annual VRE patient isolates, 886 of the 1,430 core genome loci showed missing values in at least one isolate. Overall, 57.7% of all first annual patient VRE isolates were screening isolates, while 5.9% were blood culture isolates and the remainder were other clinical samples (suppl Table 2). The proportion of screening isolates increased over time, consistent with a rise in VRE screening activity at our hospital, which intensified in response to growing VRE incidence and outbreaks. For context, the total number of VRE screening specimens increased from 384 in 2018 to 947 in 2021.

### WGS results: MLST and cgMLST analysis of the 125 VRE blood culture isolates

**Fig. 2:**
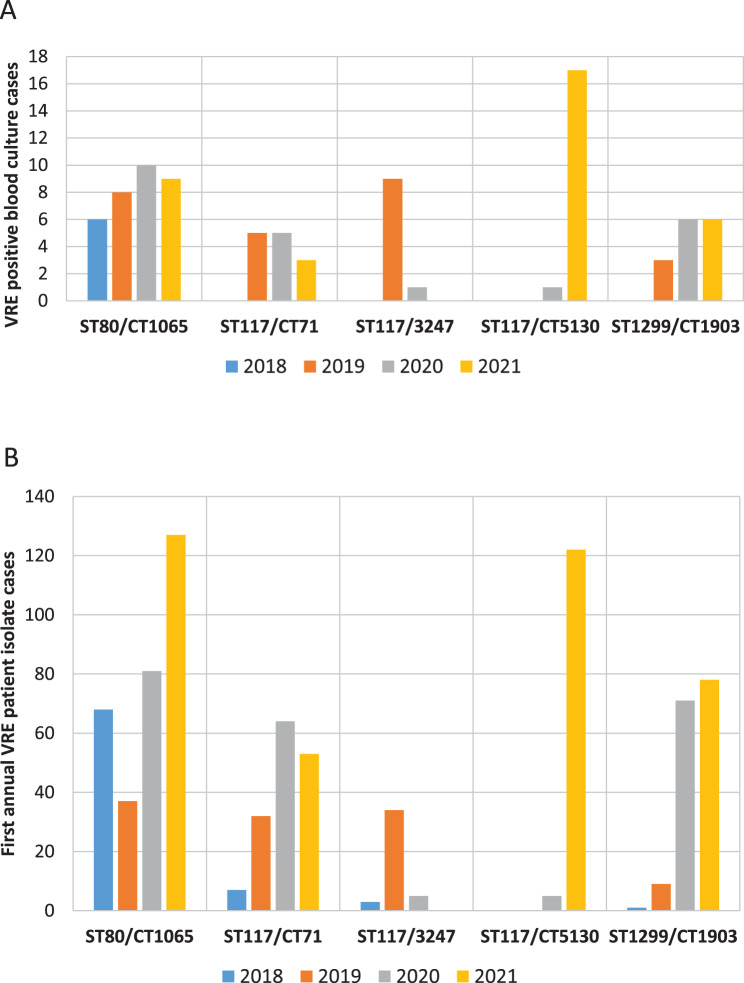
High fluctuation in the number of cases of different VRE CTs from 2018 to 2021 at university hospital regensburg **A**. within VRE BSI cases B. within first annual patient isolates. The figure illustrates the clear correlation between the number of first annual VRE isolates and VRE BSI isolates within each CT

Of the 128 VRE bloodstream infection cases identified, 125 (97.7%) isolates were available for WGS. This cohort included 69 blood culture isolates that were also first annual patient isolates, and 56 additional BSI cases where the blood culture isolate was not the first isolate of the year, yielding a total of 125 sequenced VRE blood culture isolates. Among the 125 VRE blood culture isolates, 263 of the 1,430 core genome loci had missing values. We identified nine different sequence types (STs) within these isolates. However, three different STs represented more than 90% of all isolates. Most common was ST117 (*N* = 50; 40%), followed by ST80 (*N* = 43; 34%) and ST1299 (*N* = 20; 16%). CgMLST identified 33 different CTs, but the five most common CTs represent more than two thirds of the sequenced isolates (89/125: 71.2%; details in Table 1). There was a high rate of fluctuation of different CTs over the study period (Fig. 2a).

**Table 1:** Distribution of *van* resistance genes and most common complex types of VRE blood culture isolates and first patient isolates 2018–2021 at university hospital regensburg

	VRE blood culture isolates 2018–2021 (N = 125)	VRE first patient isolates 2018–2021 (N = 1,175)	P-value
*vanA* positive isolates	36.0% (45/125)	38.5% (452/1,175)	0.63
*vanB* positive isolates	60.8% (76/125)	60.9% (715/1,175)	1.00
*vanAB* positive isolates	3.2% (4/125)	0.7% (8/1,175)	0.02*
ST17/CT900	0.8% (1/125)	0.9% (11/1,175)	1.00
ST78/CT894	1.6% (2/125)	1.3% (15/1,175)	0.67
ST80/CT1013	0	0.7% (8/1,175)	1.00
**ST80/CT1065**	**26.4% (33/125)**	**26.6% (313/1,175)**	1.00
ST80/CT2313	1.6% (2/125)	0.6% (7/1,175)	0.21
ST80/CT3322	0.8% (1/125)	1.5% (18/1,175)	1.00
**ST117/CT71**	**10.4% (13/125)**	**13.3% (156/1,175)**	0.40
ST117/CT469	2.4% (3/125)	1.4% (16/1,175)	0.42
ST117/CT2505	1.6% (2/125)	0.9% (10/1,175)	0.32
ST117/CT3247	8.0% (10/125)	3.6% (42/1,175)	0.03*
**ST117/CT5130**	**14.4% (18/125)**	**10.8% (127/1,175)**	0.23
ST721/CT3150	1.6% (2/125)	0.4% (5/1,175)	0.14
**ST1299/CT1903**	**12.0% (15/125)**	**13.5% (159/1,175)**	0.78
ST1299/CT3109	2.4% (3/125)	5.6% (66/1,175)	0.14
Other ST/CT	16.0% (20/125)	18.9% (222/1,175)	0.47

The lines representing the results for the three most common CTs among the VRE blood culture isolates and the first patient isolates are marked in bold. Values marked with a star represent statistically significant results (α ≤ 0.05).

### Distribution of CTs in VRE blood culture isolates and first patient isolates

The distribution of the most common CTs and the *van* resistance genes of VRE first patient and blood culture isolates for the whole study period is presented in Table 1 (see suppl Tables 3 a-d for the comparison of each year separately). In the analysis of the whole study period, the distribution of each CT between the VRE first patient isolates and VRE blood culture isolates is similar (Fig. 2, Table 1). However, ST117/CT3247 which was the fifth most common CT in VRE blood culture isolates with 8.0% constituted only 3.6% of all sequenced VRE first patient isolates, which reached statistical significance (*p* = 0.03). When analyzing the years separately, ST721/CT3150 was significantly more common in blood culture isolates in 2021 (*p* = 0.04), albeit with low absolute numbers. In the same year, there was also a trend towards an overrepresentation of ST117/CT5130 within the blood culture isolates (*p* = 0.08) (suppl Table 3d).

The distribution of *vanA* and *vanB* in the VRE blood culture isolates was overall similar to the distribution in the VRE first patient isolates (Table 1). The identification of both *vanA* and *vanB* in one isolate was a rare event, but significantly more common in VRE blood culture isolates (Table 1, 30.2% vs. 0.7%, *p* = 0.02).

### Clusters within VRE blood culture isolates and within the representative isolate cohort

**Fig. 3:**
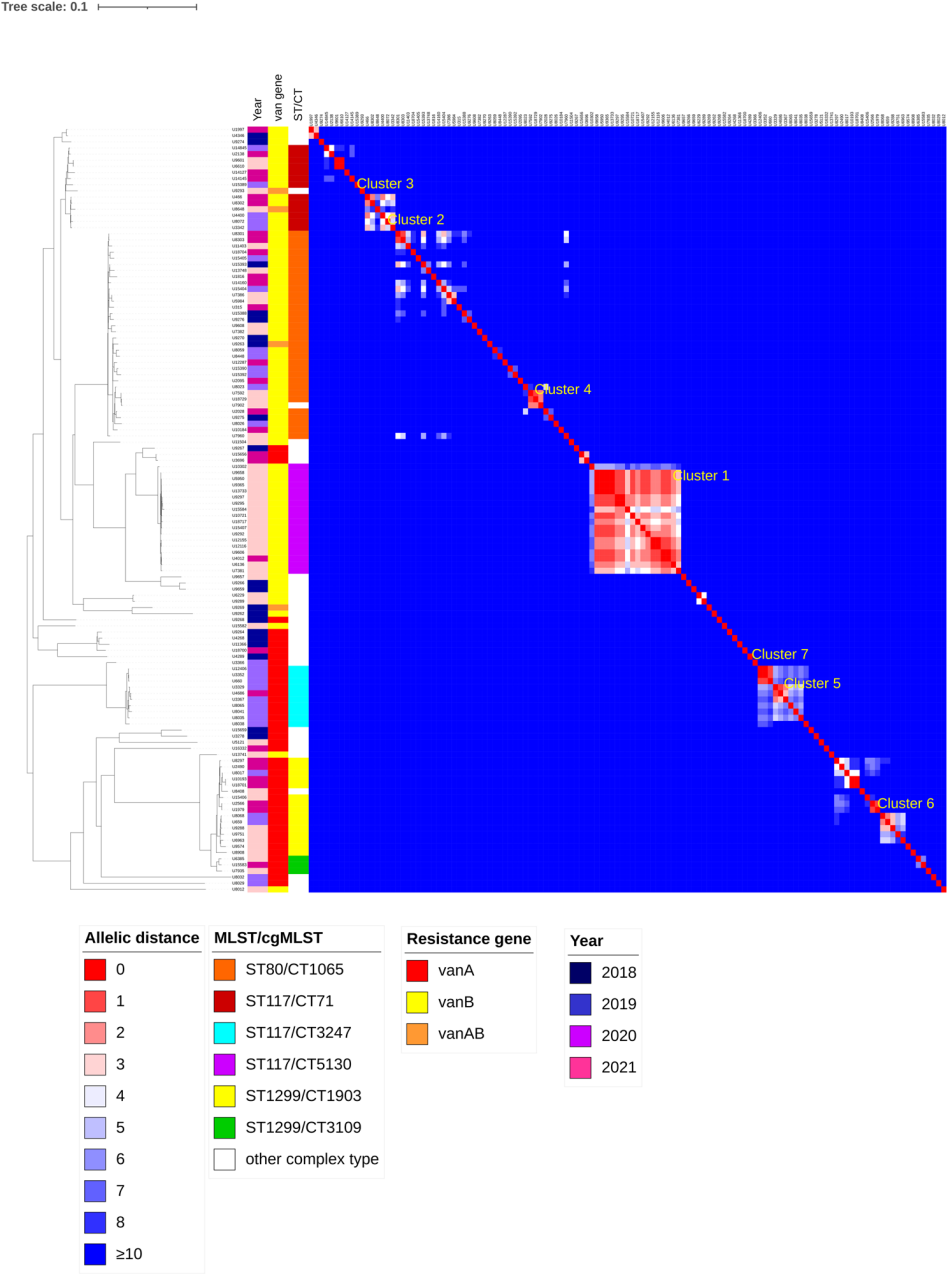
Phylogenetic tree and heatmap of cgMLST pairwise comparison of 125 VRE blood culture isolates (1,423 genes). The heatmap shows all pairwise allelic differences between isolates, with color intensity reflecting the degree of genetic similarity. Isolates are arranged according to neighbor-joining clustering and grouped by cluster affiliation. Allelic differences ≥10 are displayed in the darkest shade of blue; the maximum observed distance was 442. The phylogenetic tree is based on neighbor-joining analysis of cgMLST data exported from SeqSphere+. Color strips alongside the tree denote year of isolation, resistance gene (e.g., vanA or vanB), and MLST/cgMLST types. The seven largest clusters are labeled within the heatmap. The largest cluster, cluster 1bc (ST117/CT5130), comprises 17 isolates and stands out in size compared to other, smaller clusters. Clusters were defined using single-linkage clustering with a threshold of ≤ 3 allele differences between connected isolates. Accordingly, some clusters may include isolate pairs with > 3 allelic differences if they are indirectly connected through intermediary isolates. The heatmap format allows detailed visualization of these variations, which are not fully represented in minimum spanning tree layouts

Two separate cluster analyses were performed: one focusing exclusively on VRE blood culture isolates (*n* = 125) and a second on the representative isolate cohort (*n* = 1,147), which included all blood culture isolates and first annual patient isolates. This dual approach allowed comparison of clustering patterns between blood culture isolates and the broader hospital-wide VRE epidemiology. Pairwise cgMLST comparison affiliated 49 out of the 125 VRE blood culture isolates to 13 clusters (see Table 2). All clusters are small (2–4 isolates) except for cluster 1bc with 17 matching isolates. Figure 3 shows the genetic similarity between the 125 VRE blood culture isolates and highlights the seven largest clusters consisting of three or more isolates.

**Table 2:** Overview of clusters identified within the VRE bloodstream isolate collection (*n* = 125) based on cgMLST analysis. The table also shows how each blood culture cluster maps onto clusters identified in the representative isolate cohort (see Table 3), allowing direct comparison between clusters identified from bloodstream surveillance and those present in the broader hospital VRE population

Blood cultures only- cluster number	MLST/cgMLST	Number of patient isolates affiliated to the cluster	Number of isolates : time period of occurrence	Number of correlating cluster within the representative isolate cohort (see Table 3)
1bc	ST117/CT5130	17	14/17: Oct 2020-June 2021	2
2bc	ST80/CT1065	4	2/4: Nov 2020- Dec 2020	1
3bc	ST117/CT71	4	2/4: June 2020	9
4bc	ST80/CT1065 or CT3243	3	all: April-May 2021	7
5bc	ST117/CT3247	3	no distinct time period	5
6bc	ST1299/CT1903	3	no distinct time period	3
7bc	ST117/CT3247	3	all: May 2019- Aug 2019	5
8bc	ST117/CT2505	2	all: July 2020- Aug 2020	16 (only 8 samples, not in Table 3)
9bc	ST80/CT1065	2	all: October 2020- Feb 2021	1
10bc	ST117/CT469	2	no distinct time period	12 (only 9 isolates, not in Table 3)
11bc	ST1299/CT1903	2	all: August 2020	3
12bc	ST117/CT71	2	all: January 2021-March 2021	No additional samples
13bc	ST1299/CT1903	2	all: July 2020	23 (only 6 samples, not in Table 3)

**Table 3:** Details of the largest clusters (≥10 isolates) identified in the representative isolate cohort (*n* = 1,147), which includes all sequenced blood culture isolates together with non-redundant first annual patient isolates (see methods). The percentage of blood culture isolates in each cluster is shown, highlighting which clusters are represented by bloodstream infections and which would be missed by blood culture-based surveillance alone

Cluster number	MLST/cgMLST	Number of patients in cluster	Percentage of blood culture isolates	Mean/median/range of allele difference within specified cluster	Time period of occurrence (number of isolates)
1	ST80/CT1065	151	7.3% (11)	10.5/11/0–27	No distinct time period (Jan 2018-Dec 2021)
2	ST117/CT5130	119	14.3% (17)	1.9/2/0–7	Dec 2020-Dec 2021 (116/119)
3	ST1299/CT1903 and CT3109	100	10.0% (10)	13.1/11/0–36	July 2019-Dec 2021 (98/100)
4	ST117/CT71	39	0% (0)	3.6/4/0–9	May 2020-Dec 2021
5	ST117/CT3247	31	25.8% (8)	3.9/4/0–9	Oct 2018- Sep 2019 (30/31)
6	ST117/CT71	26	15.4% (4)	7.2/6/0–17	Jan 2019- June 2020 (23/26)
7	ST80/CT1065 and CT3243	20	15.0% (3)	2.7/3/0–7	July 2020- Dec 2021 (19/20)
8	ST1299/CT3109	20	0% (0)	2.4/2/0–7	Feb 2021- Nov 2021
9	ST117/CT71	13	38.5% (5)	2.3/2/0–6	Jan 2019- June 2020 (12/13)
10	ST1299/CT3109	13	7.7% (1)	2.8/3/0–6	Nov 2020- June 2021
11	ST80/CT1065	10	0% (0)	3.6/4/0–7	March 2020- Nov 2021

**Fig. 4:**
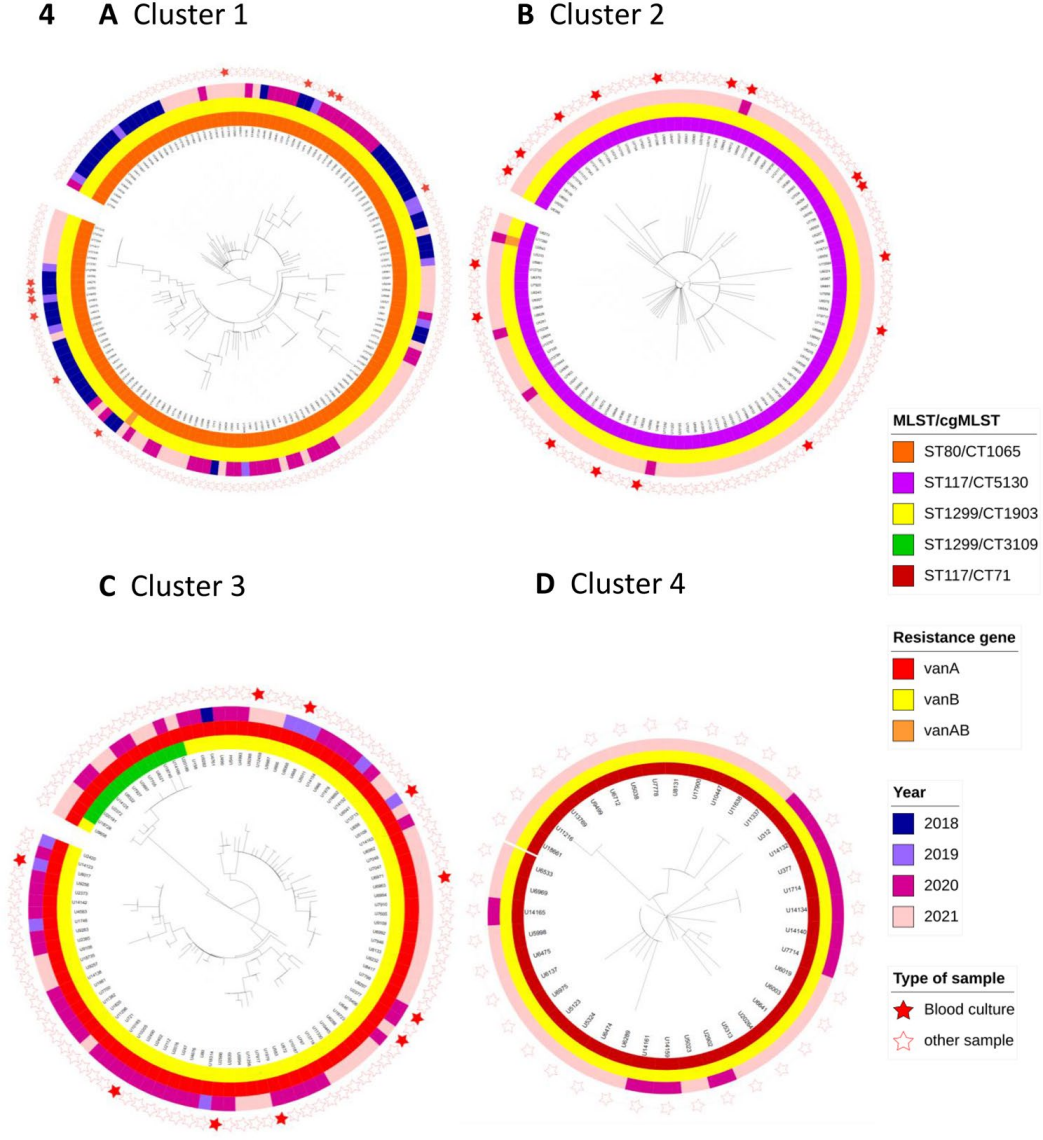
The four largest clusters with ≤3 alleles difference in cgMLST pairwise comparison within the representative isolate collection (1147 isolates) the four largest clusters are displayed as circular midpoint rooted phylogenetic trees with distance based on MLST and cgMLST alleles. The color strips of the rings represent (from inside to outside) CT, van gene and year of isolation, the red filled stars at the outer circle refer to blood culture isolates. **A**. cluster 1: ST80/CT1065, 151 isolates. **B**. cluster 2: ST117/CT5130, 119 isolates. **C**. cluster 3: ST1299/CT1903 and CT3109, 100 isolates. **D**. cluster 4: ST117, CT71, 39 isolates. This figure shows the variety of the four largest clusters in terms of the period of occurrence (e.g. isolates of cluster 1 originate from the whole study period while isolates of cluster 2 originate mostly from 2021) and the percentage of bsi cases (e.g. no bsi cases in cluster 4, several cases within cluster 2)

In the representative isolate cohort consisting of 1,147 VRE isolates (125 sequenced blood culture isolates and 1,022 non-redundant first annual patient isolates), 834 isolates (72.7%) were grouped into 98 clusters, while the remainder were singletons. Among these, 11 large clusters comprising 10 or more isolates were identified (Table 3; Fig. 4, suppl Fig. 1). The duration of each cluster is also reported in Table 3, showing the collection time frames to illustrate temporal clustering patterns.The three largest clusters within this isolate cohort comprising ≥100 isolates are ST80/CT1065, ST117/CT5130 and ST1299/CT1903 and CT3109 (Table 3, Fig. 4). The percentage of blood culture isolates within the 11 largest clusters ranged from 0 to 38.5%, so some of these clusters might not be represented when analysing the blood culture isolates only (e.g. cluster 4: Fig. 4d, suppl Fig. 1).

To further contextualize the clustering analysis, we examined the distribution of pairwise cgMLST allelic distances in the representative isolate cohort (suppl Fig. 2). Within the four largest clusters, distance distributions varied substantially (suppl Fig. 3). Stratified analyses showed that low allelic differences were observed only within sequence types but not between them (suppl Fig. 4), and that intra-cluster distances were markedly lower than inter-cluster distances (suppl fig 5).

## Discussion

By utilizing WGS on VRE blood culture and annual first patient isolates our study sheds light on the increase of VRE BSIs at our institution from 2018 to 2021. We identified a rising number of VRE carriers caused by several large-scale VRE outbreaks as triggers for this development. Distribution of CTs within VRE blood culture isolates and first annual patient isolates was generally similar. Still, there are hints that certain CTs or sets of *van* genes imply a higher risk for VRE BSIs.

In our hospital, the increase in the VRE incidence density parallels the rise in VRE BSI rates. For VRE carriers, a recent meta-analysis estimated the risk for VRE infection to be 8% within 30 days follow-up [13]. However, patient risk factors such as underlying medical conditions, age, immunosuppression, and antibiotic exposure play a critical role in the likelihood of developing VRE BSI [14–16]. In addition, genomic data support that colonization often precedes infection: Rubin et al. demonstrated that in 13 out of 19 patients with both colonization and invasive isolates, the VRE strains were genetically identical based on cgMLST analysis [17].

Our results show that isolates affiliated with clusters within the representative isolate cohort were mainly collected within a short and defined period, suggesting transmission events that led to several outbreaks and contributed to the rise in the VRE incidence rate at our institution. Although not the focus of this study, our findings support continued caution regarding the relaxation of infection control measures in high-risk settings [18].

While temporal trends within clusters were analyzed to support the presence of transmission events (as shown in Table 3), a spatial (ward-level) analysis of cluster distribution was not performed. As the primary aim of our study was to assess the representativeness of VRE blood culture isolates and to identify broader epidemiological patterns, we did not conduct a detailed spatial analysis or reconstruction of transmission chains. For some clusters, independent epidemiological investigations confirmed outbreak events within specific hospital departments (data not shown), but these analyses are not included in the present manuscript.

While many clusters showed low intra-cluster allelic differences and temporal clustering consistent with recent transmission events, this pattern was not universal. For example, cluster 1 (ST80/CT1065) in the representative isolate cohort shows a high mean number of allele differences between isolates and an absence of a pronounced collection time window (Table 3, suppl Fig. 3). This suggests that some clusters might, at least partly, represent endemic circulation clones. In line with this, for ST80/CT1065 a prominent role in Bavarian hospitals has been described before [19].

In addition to nosocomial transmission, recent studies suggest that the expansion of specific VRE lineages may also be driven by intrinsic biological advantages such as bacteriocin production. Notably, emergent lineages such as ST80 and ST117 have been shown to frequently encode bacteriocin T8 (also known as bac43 or hiracin JM79), which confers a competitive advantage in colonization and enables these strains to outcompete other *E. faecium* in vitro and in the mammalian gut [20, 21]. These bacteriocin-producing strains have been implicated in lineage replacement events both locally and globally. In our cohort, the marked increase in VRE BSIs in 2021 coincided with the emergence of ST117/CT5130, a sublineage of ST117. While we did not assess bacteriocin gene carriage in our isolates, the association of ST117 with bacteriocin T8 in other settings raises the possibility that this mechanism may also have contributed to clonal expansion at our institution.

These considerations, along with the observed VRE clusters, must also be interpreted in the context of the burden posed by the COVID-19 pandemic. As a tertiary care and extracorporeal membrane oxygenation (ECMO) center, treating a large number of patients with severe COVID-19 acute respiratory distress syndrome resulted in a high workload for the medical staff and necessitated significant organizational changes, including the restructuring of ICUs. The negative effect of understaffing for the infection risk for patients has been described and is likely associated with a lower compliance with infection control measures [22]. Furthermore, reports of VRE outbreaks within patients isolated under COVID-19 precautions, which included both respiratory and contact precautions [23, 24], have been published.

Some VRE strains like ST796, ST1299, ST117/CT71 have gained attention due to their fast spread within countries or even beyond borders [6, 25–28]. However, the question of whether there are VRE high-risk clones not only in terms of transmission but also in terms of their potential for invasiveness has not yet been answered.

We found that strains commonly colonizing patients or causing other clinical infections are mostly also those causing BSIs. In particular, we did not find an overrepresentation of the above mentioned ST1299 and ST117/CT71 in VRE BSIs. However, some CTs like ST117/CT3247 were significantly more common in blood culture isolates, albeit with low absolute numbers. Another interesting finding is that while *vanA* and *vanB* positive isolates were represented with similar frequency in both groups, the rare cases where both *vanA* and *vanB* genes are found within the same isolate were more commonly associated with BSI. This may reflect greater illness severity and antibiotic exposure among patients with VRE BSI, as suggested in studies in HSCT patients and in hospital surveillance data [14–16], potentially making their isolates more prone to acquiring additional resistance determinants. Comprehensive resistance gene profiling beyond *vanA* and *vanB* was not performed, as the detection of other clinically relevant mechanisms (e.g., for linezolid and daptomycin) is incomplete in the tool we used.

Strengths of our study include the extensive WGS data from both VRE blood culture isolates and annual first patient isolates, providing a comprehensive overview of the whole VRE epidemiology at our institution. This is the first study to systematically compare these two groups with a high number of included isolates. Limitations include its single-center design, which may restrict generalizability, the varying intensity of VRE screening during the study period, and incomplete core genome coverage in some isolates. In addition, we used cgMLST, which, while standardized and scalable, offers lower resolution than SNP-based approaches. Finally, the use of a single-linkage approach for cluster definition may have inflated cluster size by grouping together isolates without strong epidemiological or genotypic linkage, as illustrated by the distribution of pairwise allelic distances (suppl fig 2–5). In addition, our focus on transmission may have overlooked other bacterial factors contributing to VRE expansion, such as colonization traits, antibiotic tolerance, or bacteriocin production.

Overall, our findings support with certain restrictions the strategy of sequencing VRE blood culture isolates only to gain an overview of VRE epidemiology in a single institution, particularly in resource-limited settings. However, this approach may miss certain transmission clusters involving little or no BSIs. Ideally, larger, multicenter and international studies should validate our findings and assess their generalizability.

## Electronic supplementary material

Below is the link to the electronic supplementary material.

Supplementary material 1

Supplementary material 2

Supplementary material 3

Supplementary material 4

Supplementary material 5

Supplementary material 6

## Data Availability

The datasets generated and analyzed during the current study are available in the National Center for Biotechnology Information (NCBI) repository: https://www.ncbi.nlm.nih.gov/bioproject/PRJNA1210141; https://www.ncbi.nlm.nih.gov/bioproject/PRJNA1210857; https://www.ncbi.nlm.nih.gov/bioproject/PRJNA1211302; https://www.ncbi.nlm.nih.gov/bioproject/PRJNA1211329; https://www.ncbi.nlm.nih.gov/bioproject/PRJNA1211508; https://www.ncbi.nlm.nih.gov/bioproject/PRJNA1212862

## Abbreviations

VRE: Vancomycin-resistant *Enterococcus faecium*
BSI: Bloodstream Infection
WGS: Whole Genome Sequencing
MLST: Multi-Locus Sequence Typing
cgMLST: core-genome Multi-Locus Sequence Typing
CT: Complex Type
VSE: Vancomycin-susceptible *Enterococcus faecium*
ICU: Intensive Care Unit
PCR: Polymerase Chain Reaction
MALDI-TOF MS: Matrix-Assisted Laser Desorption Ionization Time-of-Flight Mass Spectrometry
ECMO: Extracorporeal Membrane Oxygenation
