# Supplementary figures and images for: The potential of genome-oriented blood culture surveillance of vancomycin-resistant enterococcus faecium (VRE) to mirror local VRE epidemiology: a retrospective analysis and systematic comparison of VRE blood culture and VRE first patient isolates
Source: BMC Infect Dis. 2025 Nov 28;25:1685. doi: 10.1186/s12879-025-12183-9 (PMC12670834; doi:10.1186/s12879-025-12183-9)

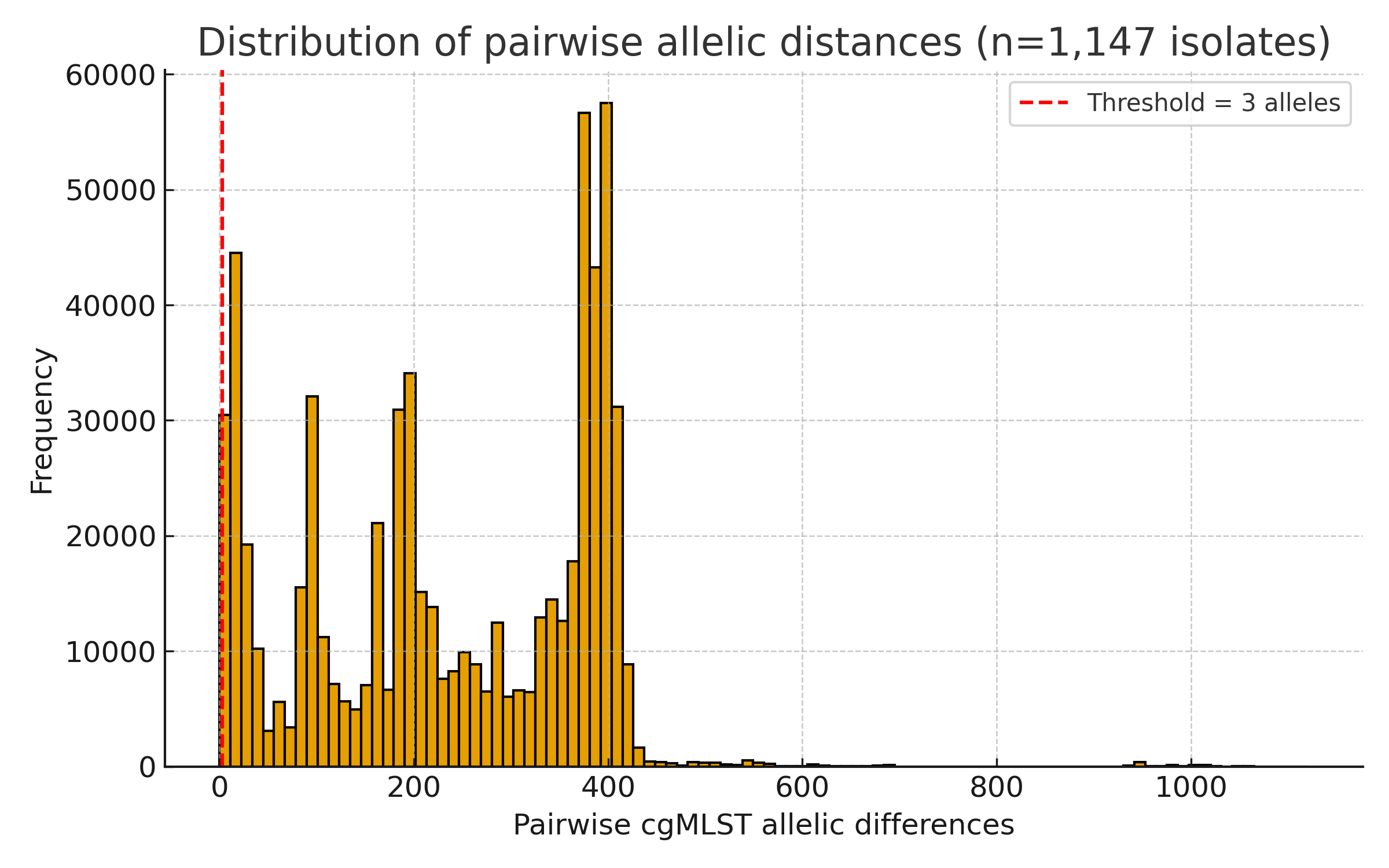

Supplement: Supplementary file 2 — Supplementary material 2 [file 12879_2025_12183_MOESM2_ESM.png]

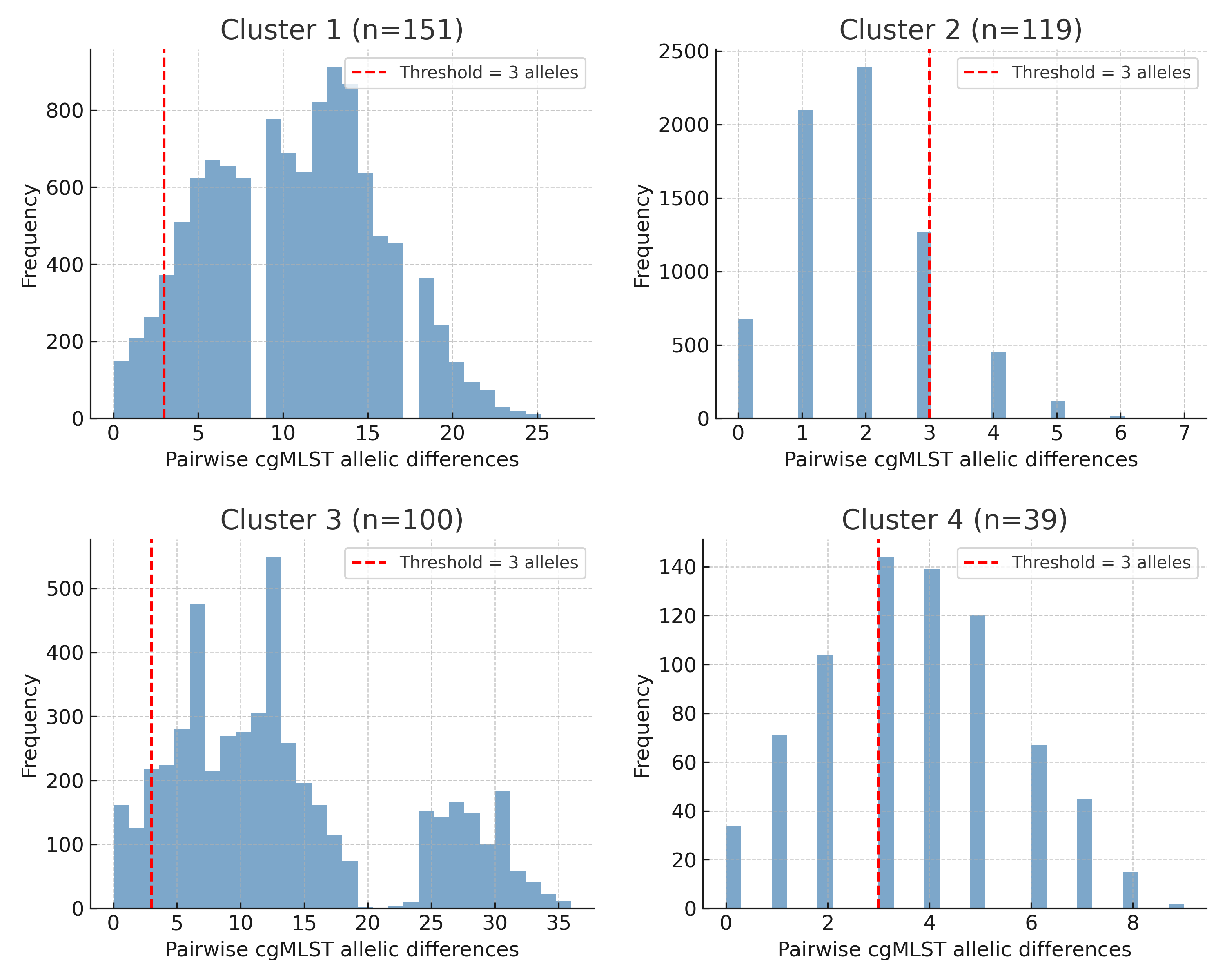

Supplement: Supplementary file 3 — Supplementary material 3 [file 12879_2025_12183_MOESM3_ESM.png]

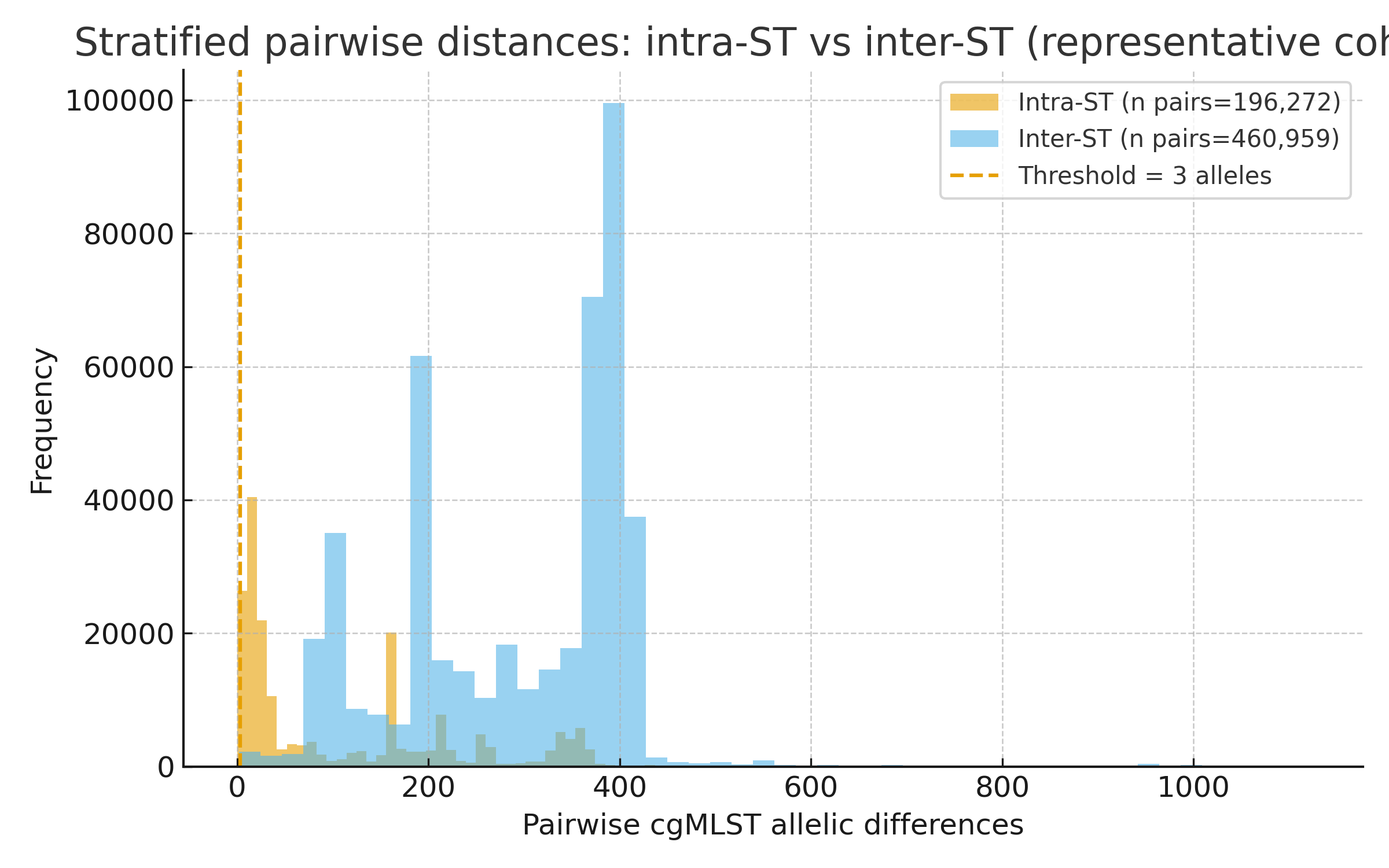

Supplement: Supplementary file 4 — Supplementary material 4 [file 12879_2025_12183_MOESM4_ESM.png]

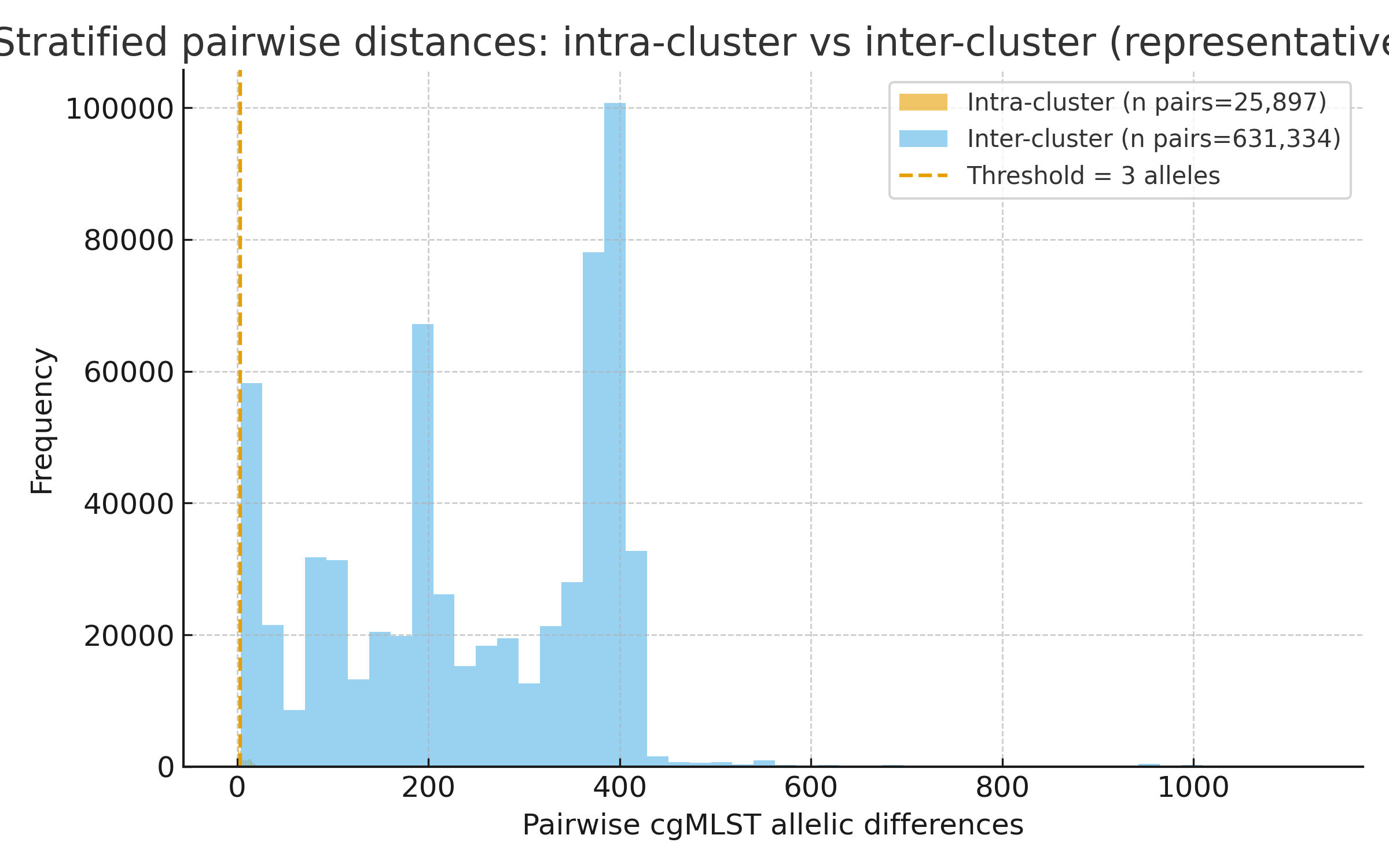

Supplement: Supplementary file 5 — Supplementary material 5 [file 12879_2025_12183_MOESM5_ESM.png]
