# Supplementary material for: The potential of genome-oriented blood culture surveillance of vancomycin-resistant enterococcus faecium (VRE) to mirror local VRE epidemiology: a retrospective analysis and systematic comparison of VRE blood culture and VRE first patient isolates
Source: BMC Infect Dis. 2025 Nov 28;25:1685. doi: 10.1186/s12879-025-12183-9 (PMC12670834; doi:10.1186/s12879-025-12183-9)
Supplement: Supplementary file 6 — Supplementary material 6 [file 12879_2025_12183_MOESM6_ESM.docx]

**Supplementary tables and figure**

**Suppl Table 1: Illustration of inclusion criteria of isolates for the representative isolate cohort**

| Patient | Date of isolation of VRE specimen | material | CT | Inclusion in representative isolate cohort? | Reason for inclusion/exclusion |
| --- | --- | --- | --- | --- | --- |
| a | 01.03.2019 | urine | 1065 | yes | First annual patient isolate in the study period |
| a | 22.01.2020 | urine | 3350 | yes | Subsequent first annual patient isolate with differing CT to the already included isolate |
| a | 25.05.2021 | rectal screening | 3350 | no | Subsequent first annual patient isolate with identical CT to an already included isolate |
|  |  |  |  |  |  |
| b | 11.02.2020 | rectal screening | 71 | yes | First annual patient isolate in the study period |
| b | 06.04.2021 | rectal screening | 1903 | yes | Subsequent first annual patient isolate with differing CT to the already included isolate |
|  |  |  |  |  |  |
| c | 17.05.2018 | wound swab | 469 | yes | First annual patient isolate in the study period differing from the CT of the blood culture |
| c | 10.03.2020 | blood culture | 1065 | yes | First patient blood culture |
|  |  |  |  |  |  |
| d | 05.03.2020 | rectal screening | 1903 | no | First annual patient isolate in the study period but identical with the CT of the blood culture isolate |
| d | 13.03.2020 | blood culture | 1903 | yes | First patient blood culture isolate |
|  |  |  |  |  |  |
| e | 06.05.2021 | bile | 1065 | initially yes* | First annual patient isolate in the study period differing from the CT of the blood culture* |
| e | 17.05.2021 | blood culture | 3243 | yes | First patient blood culture isolate |

* This isolate was eventually excluded: In the cluster analysis of all isolates of the representative isolate cohort both of the isolates of patient e (bile and blood culture) belonged to cluster 7 (ST80/CT1065 and CT3243. These two isolates have only 3 alleles difference in pairwise cgMLST comparison in spite of belonging to two different CTs. In order to avoid artificial clustering, the isolate stemming from bile was consequently excluded from the representative isolate cohort.

**Suppl Table 2: Details about the VRE first annual patient isolates**

| Year | VRE first patient isolates | Number of successfully sequenced first patient isolates | Proportion of screening isolates from successfully sequenced first patient isolates (absolute numbers (percentage)) | Proportion of blood culture isolates from successfully sequenced first patient isolates (absolute numbers (percentage)) |
| --- | --- | --- | --- | --- |
| 2018-2021 | 1,534 | 1,175 (76.6%) | 678 (57.7%) | 69 (5.9%) |
| 2018 | 293 | 178 (60.8%) | 102 (57.3%) | 11 (6.2%) |
| 2019 | 307 | 156 (50.8%) | 59 (37.8%) | 19 (12.2%) |
| 2020 | 356 | 318 (89.3%) | 159 (50.0%) | 17 (5.3%) |
| 2021 | 578 | 523 (90.5%) | 358 (68.5%) | 22 (4.2%) |

**Suppl Table 3a:** Distribution of *van* resistance genes and most common CTs of VRE blood culture isolates and first patient isolates in 2018 at University Hospital Regensburg

|  | VRE blood culture isolates in 2018  (N=20) | VRE first patient isolates in 2018  (N=178) | P-value |
| --- | --- | --- | --- |
| *vanA* positive isolates | 40.0% (7/20) | 33.7% (60/178) | 1.00 |
| *vanB* positive isolates | 50.0% (10/20) | 65.7% (117/178) | 0.22 |
| *vanAB* positive isolates | 10.0% (2/20) | 0.6% (1/178) | 0.03* |
| ST17/CT900 | 5.0% (1/20) | 4.5% (8/178) | 1.00 |
| **ST78/CT894** | **10.0% (2/20)** | **5.1% (9/178)** | **0.31** |
| ST80/CT1013 | 0 | 3.9% (7/178) | 1.00 |
| **ST80/CT1065** | **30.0% (6/20)** | **38.2% (68/178)** | **0.63** |
| **ST80/CT2313** | **10.0% (2/20)** | **2.8% (5/178)** | **0.15** |
| ST117/CT71 | 0 | 3.9% (7/178) | 1.00 |
| **ST117/CT469** | **10.0% (2/20)** | **3.4% (6/178)** | 0.19 |
| Other ST/CT | 35.0% (6/20) | 38.2% (68/178) | 0.63 |

The lines representing the results for the most common CTs among the VRE blood culture isolates and the first patient isolates are marked in bold. Values marked with a star represent statistically significant results (α ≤ 0.05).

**Suppl Table 3b:** Distribution distribution of *van* resistance genes and most common CTs of VRE blood culture isolates and first patient isolates in 2019 at University Hospital Regensburg

|  | VRE blood culture isolates in 2019  (N=28) | VRE first patient isolates in 2019  (N=156) | P-value |
| --- | --- | --- | --- |
| *vanA* positive isolates | 53.6% (15/28) | 48.7% (76/156) | 0.69 |
| *vanB* positive isolates | 46.4% (13/28) | 49.4% (77/156) | 0.84 |
| *vanAB* positive isolates | 0 | 1.9% (3/156) | 1.00 |
| **ST80/CT1065** | **28.6% (8/28)** | **23.7% (37/156)** | **0.63** |
| ST80/CT3322 | 0 | 7.1% (11/156) | 0.22 |
| **ST117/CT71** | **17.9% (5/28)** | **20.5% (32/156)** | **1.00** |
| **ST117/CT3247** | **32.1% (9/28)** | **21.8% (34/156)** | **0.23** |
| **ST1299/CT1903** | **10.7% (3/28)** | **5.8% (9/156)** | **0.40** |
| Other ST/CT | 10.7% (3/28) | 21.1% (33/156) | 0.30 |

The lines representing the results for the most common CTs among the VRE blood culture isolates and the first patient isolates are marked in bold. Values marked with a star represent statistically significant results (α ≤ 0.05).

**Suppl Table 3c:** Distribution of *van* resistance genes and most common CTs of VRE blood culture isolates and first patient isolates in 2020 at University Hospital Regensburg

|  | VRE blood culture isolates in 2020  (N=29) | VRE first patient isolates in 2020  (N=318) | P-value |
| --- | --- | --- | --- |
| *vanA* positive isolates | 41.4% (12/29) | 43.4% (138/318) | 1.00 |
| *vanB* positive isolates | 58.6% (17/29) | 56.3% (179/318) | 0.85 |
| *vanAB* positive isolates | 0 | 0.3% (1/318) | 1.00 |
| **ST80/CT1065** | **34.5% (10/29)** | **25.5% (81/318)** | **0.28** |
| **ST117/CT71** | **17.2% (5/29)** | **20.1% (64/318)** | **0.81** |
| ST117/CT2505 | 6.9% (2/29) | 2.5% (8/318) | 0.20 |
| ST117/CT3247 | 3.4% (1/29) | 1.6% (5/318) | 0.41 |
| ST117/CT5130 | 3.4% (1/29) | 1.6% (5/318) | 0.41 |
| **ST1299/CT1903** | **20.7% (6/29)** | **22.3% (71/318)** | **1.00** |
| ST1299/CT3109 | 3.4% (1/29) | 3.8% (12/318) | 1.00 |
| Other ST/CT | 10.3% (3/29) | 22.6% (72/318) | 0.16 |

The lines representing the results for the most common CTs among the VRE blood culture isolates and the first patient isolates are marked in bold. Values marked with a star represent statistically significant results (α ≤ 0.05).

**Suppl Table 3d:** Distribution of *van* resistance genes and most common CTs of VRE blood culture isolates and first patient isolates in 2021 at University Hospital Regensburg

|  | VRE blood culture isolates in 2021  (N=48) | VRE first patient isolates in 2021  (N=523) | P-value |
| --- | --- | --- | --- |
| *vanA* positive isolates | 20.8% (10/48) | 34.0% (178/523) | 0.08 |
| *vanB* positive isolates | 75% (36/48) | 65.4% (342/523) | 0.20 |
| *vanAB* positive isolates | 4.2% (2/48) | 0.6% (3/523) | 0.06 |
| **ST80/CT1065** | **18.8% (9/48)** | **24.3% (127/523)** | **0.48** |
| **ST117/CT71** | **6.3% (3/48)** | **10.1% (53/523)** | **0.61** |
| **ST117/CT5130** | **35.4% (17/48)** | **23.3% (122/523)** | **0.08** |
| **ST1299/CT1903** | **12.5% (6/48)** | **14.9% (78/523)** | **0.83** |
| **ST1299/CT3109** | **4.2% (2/48)** | **10.3% (54/523)** | **0.21** |
| ST721/CT3150 | 4.2% (2/48) | 0.4% (2/523) | 0.04* |
| Other ST/CT | 18.8% (9/48) | 16.6% (87/523) | 0.69 |

The lines representing the results for the most common CTs among the VRE blood culture isolates and the first patient isolates are marked in bold. Values marked with a star represent statistically significant results (α ≤ 0.05).

**Suppl. Figure 1:**

Minimum spanning tree (MST) for 1,147 isolates from the representative isolate cohort, based on pairwise allele comparison using MLST (7 genes) and cgMLST (1,423 genes). VRE blood culture isolates are marked in red; non-blood culture first patient isolates are shown in light blue. Clusters are defined by ≤3 allelic differences. Clusters containing ≥10 isolates are shaded in gray.

This figure places all blood culture isolates in the context of the broader hospital VRE epidemiology and illustrates their distribution across major clusters. The representative isolate cohort was constructed to avoid artificial clustering by including only one isolate per patient per complex type (CT), with inclusion rules described in the Methods and Supplementary Table 1. This approach ensures a non-redundant yet comprehensive overview of VRE population structure and cluster composition across isolate types.

**Suppl. Figure 2:**

Distribution of pairwise cgMLST allelic distances among all isolates of the representative isolate cohort (n = 1,147).

The histogram shows 657,231 unique pairwise comparisons. The dashed red line marks the cluster definition threshold (≤3 alleles). Summary statistics: median 234 alleles (IQR 144–324), mean 238, maximum 1,121.

**Suppl. Figure 3:**

Within-cluster pairwise cgMLST allelic distance distributions for the four largest clusters of the representative isolate cohort.

Histograms display all unique pairwise comparisons per cluster; the dashed red line marks the cluster definition threshold (≤3 alleles).

Cluster 1: n = 151 isolates, 11,325 pairwise comparisons; median 11 alleles (range 0–27).

Cluster 2: n = 119 isolates, 7,021 pairwise comparisons; median 2 alleles (range 0–7).

Cluster 3: n = 100 isolates, 4,950 pairwise comparisons; median 11 alleles (range 0–36).

Cluster 4: n = 39 isolates, 741 pairwise comparisons; median 4 alleles (range 0–9).

**Suppl. Figure 4:**

Stratified pairwise cgMLST distance distributions by sequence type.

Histogram contrasting intra-ST vs. inter-ST comparisons in the representative isolate cohort (n = 1,147; 657,231 pairwise comparisons). The dashed red line marks the cluster definition threshold (≤3 alleles). Median (IQR) intra-ST distances: 39 (167); inter-ST: 339 (198). The proportion of comparisons ≤3 alleles was 4.8% intra-ST vs. 0.002% inter-ST.

**Suppl. Figure 5:**

Stratified pairwise cgMLST distance distributions by cluster membership (single-linkage, threshold ≤3 alleles).

Histogram contrasting intra-cluster vs. inter-cluster comparisons across 98 clusters (≥2 isolates) identified in the representative isolate cohort (n = 1,147). The dashed red line marks the cluster definition threshold (≤3 alleles). Median (IQR) intra-cluster distances: 6 (11); inter-cluster: 255 (246). The proportion of comparisons ≤3 alleles was 36.1% intra-cluster vs. 0% inter-cluster.
